# Why few older adults participate in complex motor skills: a qualitative study of older adults’ perceptions of difficulty and challenge

**DOI:** 10.1186/s12889-015-2501-z

**Published:** 2015-11-26

**Authors:** Katarina P. Kraft, Kylie A. Steel, Freya Macmillan, Rebecca Olson, Dafna Merom

**Affiliations:** School of Science and Health, University of Western Sydney, Penrith, NSW 2751 Australia; School of Social Science, The University of Queensland, St Lucia, 4072 Australia

**Keywords:** Older adults, Physical activity, Focus groups, Gentile’s taxonomy, Complex motor skills

## Abstract

**Background:**

Maintaining neuromotor fitness across the life course is imperative. It can reduce falls in older individuals and improve/maintain physical and cognitive functioning. Complex motor skills (CMS) are involved in many physical activities (e.g., ball games, dance), which can improve neuromotor fitness. However, few older adults participate in CMS. This study aimed to understand how older adults perceive the degree of difficulty and challenge, using Gentile’s taxonomy of motor skills as a framework.

**Methods:**

Six focus groups (FGs) were conducted with older adults (aged 61–92 years; *N* = 36) using a semi-structured question guide, to explore older adults’ perceptions of difficulty and challenges associated with physical activity types. FGs were conducted in three villages and community groups in Sydney, Australia. Verbatim transcripts were coded inductively following a grounded theory approach to analysis to discover categories and concepts based on participants’ views.

**Results:**

Older adults perceived physical effort and pace as influencing difficulty where as challenging activities were not found to hinder older adults’ willingness to participate. Other challenges in performing activities were attributed to: skill level, environment conditions (e.g., pool versus ocean swimming) and variations influencing complexity. Social and interpersonal issues, such as embarrassment, rapport with instructors, prior experience/ familiarity, in addition to physical effort, were other central features of older adults’ perceptions of physical activities. Themes that appeared to increase the likelihood of participation in CMS were: age appropriate modification; enjoyment; social aspects; past experience; and having experienced instructors.

**Conclusions:**

This study offers recommendations for increasing participation in CMS. Modifying activities to suit ability and age and increasing exposure during the life span may help maintain participation into old age. Gentile’s taxonomy provides an appropriate framework for classifying activities as simple or complex, which were recognised by participants on a descriptive level. Existing and new sports, which have been modified for old age, should be made available to older adults. Within the motor learning literature, the focus on older adults is limited. If activity complexity translates to improved cognitive abilities as well as improved individual neuromotor performance, the challenge of modifying activities to suit older adults’ preferences needs to be addressed.

## Background

Over 3.0 million people in Australia are currently aged ≥65 years, and this number is projected to increase in Australia and globally [1–3]. With ageing, for those who survive or are unaffected by chronic diseases, the greatest health threat is neurophysiological and cognitive decline, which increases the risk of falls, injuries and dementia [4, 5]. Delaying the onset of cognitive decline and risk of falls even by one year can reduce the amount of hardship placed on individuals and families, as well as the medical costs associated with care of people in this age group [6, 7].

Physical activity and exercise programs have been shown to assist physiological capacities towards healthy ageing, as extensively summarised by the American College of Sport Medicine (ACSM) [8]. Compared to previous recommendations (2009), the most recent ACSM report (2011) places greater emphasis on the inclusion of neuromotor fitness (i.e., balance, agility, coordination and reaction time: for athletes these are considered as performance indicators). Whereas for older adults improvements in neuromotor fitness components may improve or maintain their physical functioning [8]. Neuromotor fitness components incorporate a variety of motor skills, often used in a range of physical activities. As opposed to simple motor skills, which are more predictable involving less variability (goal-directed; e.g., running and throwing). Complex motor skills (CMS) require significantly higher levels of intricately (organisation/components) coordinated body movements requiring learning and practice hence incorporating greater neuromotor fitness components. CMS cannot be mastered in a single practice session as they involve unpredictable and changing environments where the person and/or object are in motion, [9] such as ball sports, dance, tai chi, and martial arts. Hence any reference to CMS will consider neuromotor fitness components to be involved in those activities.

Cross-sectional comparative studies have indicated that athletes generally demonstrate superior balance ability compared with non-athletes of a similar age [10]. Similarly, long-term participation in sport by older adults was found to be associated with better neuromotor capacities compared to older adults with no record of participation in sport [11]. A small number of comparative studies have also shown such advantages persist to old age; for example, long term dancers had better gait and balance abilities than age-matched non-dancers [12]. Furthermore, intervention research shows that participation in complex motor activity, such as tai chi, results in better measures of fitness overall, including balance, compared to brisk walking [13]. Tai chi interventions have also been found to result in superior balance and cognitive performances compared to participation in western exercise classes. The significant and novel cognitive improvements (found from the digit-span tests which consider indicators of attention, concentration, and mental tracking), in tai chi participants compared to western exercise group participants, may be due to the unique emphasis on concentration in movements found in tai chi [14].

Existing literature appears to show potential for increased health benefits through specific activity types. Public health research has given little attention to the advantages of specific activity types that are cognitively challenging and more complex in their execution. If pilot studies continue to show positive impacts on a variety of functional domains as well as physical and cognitive functioning improvements, this calls for further research on health benefits that apply to CMS, such as golf, ball sports and aquatic sports. Prior to addressing benefits of CMS, population-based research shows that only one in five older adults will take up any sport [15]. Walking and lifestyle physical activity, such as gardening and household chores, are the most common activities in which older adults participate [15]. Hence an understanding of what shapes older adults’ perceptions of difficulties or barriers to participate in CMS is needed first to facilitate the development of effective and tailored physical activity programs (whether intervention or community driven), in order to increase participation.

Despite having several taxonomies to interpret activity difficulty and challenges such as those offered by Schmidt RA [16] or Gentile’s [17], limited research in the field of movement science and sport acquisition focus on older adults. With motor performance beginning to deteriorate at some stage in older adulthood [18] and few older adults taking up any sport [15], this study therefore sought to understand how older adults perceive the degree of difficulty on a range of physical activities in order to understand how their perceptions may influence participation. A qualitative approach was chosen, focusing on exploring perceptions and attitudes from an insider’s perspective [19]. Gentile’s taxonomy is used as a framework in the discussion, providing a comparison to any further findings from older adults’ perspectives that are not yet considered in such a comprehensive platform.

## Methods

Focus group (FG) discussions were conducted by the lead researcher with English speaking participants aged ≥60 years, living independently in the community and in retirement villages in the Greater Sydney region. Participants were recruited from non-physical activity contexts, such as bingo sessions rather than lawn bowl or dancing groups, to minimise extreme variation in perceptions across groups. FGs were held at a familiar location to participants (i.e., village, local cafe) to foster feelings of comfort amongst participants, which can be important to establishing rapport [20]. The study was approved by the University of Western Sydney (HREC #H10221).

FGs were conducted using a semi-structured discussion guide (Table 1), to provide consistency in the questions delivered to groups and to allow the moderator sufficient flexibility to focus on issues salient to those under study [19, 20]. Discussion guides were developed in accordance with the co-researchers for this study, who have expertise in physical activity participation in older adults and in acquisition of motor skills. When development was completed, the question guide was tested within and revised by the co-researchers, as well as being pilot-tested in a group of ten older adults living in a retirement village.

**Table 1:** Focus group physical activity question guide

1. In what physical activities do you think older adults currently participate?	
2. From the listed activities (shown below) place in order: a. Easy to Difficult (scale 1–10, 1 being easiest) b. Least challenging to most challenging (Focus on level of skill involved in the activity and dismiss physical effort)	
Key activities	Extra activities
• Walking • Fishing • Swimming • Dancing • Gym workout • Aerobics calisthenics • Lawn bowls • Golf • Cycling • Racquet sports	• Tai chi • Running and jogging • Weightlifting • Water aerobics • Zumba • Yoga • Rowing • Sailing • Table tennis
3. In each life stage which physical activities did you previously participate in? Combine with next question (influences) • Childhood (6–12 years) • Adolescent (12–18 years) • Young adult (18–30) • Working age (30–50) • Older adult (50+) a. What do you think influenced your physical activity choices in each of these periods of your life?	
4. If offered, what is the likelihood that you would participate in the listed activities? Are there any you would be more or less inclined to participate in? Why? a. Are there any barriers associated with participating in these activities? What would stop you from participating? Why? b. What are the benefits of each activity?	

The study recognised that gender differences in relation to physical activity may be apparent [15, 21]. Initially separate FGs were planned. However, after the initial mixed-gender groups for the pilot study, it became apparent that discussions were open, with both males and females freely providing input in discussions, allowing comparisons across genders. Six FGs were planned to allow for a range of perspectives to be collected. Based on the research team’s previous experience, this was deemed a sufficient number of discussions to reach data saturation [22].

Sampling was purposive, intending to collect a variety of opinions and allow variation between participants [19, 23, 24]. All FGs were scheduled by sending emails to retirement village managers and approaching administration and community groups in the Greater Sydney region. Prospective participants were informed about study objectives, prior to signing consent forms.

### Framework used to assess motor skills difficulties and challenge

In the field of movement science, a number of classification systems can be used to determine the degree of complexity for physical activities such as one-dimensional systems, e.g., open vs. closed skills, and gross vs. fine [16]. However these systems are limited and do not provide the depth often required in a day-to-day setting. Therefore in order to best interpret older adults’ perceptions of difficulties involved in a range of physical activities a two-dimensional system should be used. Gentile (1972) offers the most comprehensive system for classification with a 16-item taxonomy designed to include a variety of movement characteristics. This incorporates several factors including environmental context, object manipulation and the body movement status of the individual components.

Specifically, Gentile’s taxonomy is a skill progression platform consisting of two main perspectives, environment context and action function. The environment context is sub divided into a closed environment (stationary regulatory) or open environment (in-motion regulatory) combined with either the performance condition remaining constant (no-intertrial variability) or differing each trial (intertrial variability). This is then combined with action function, which considers body stability or body movement in combination with object manipulation or no object manipulation. Environment context and action function combine to provide a logical sequence and progression of skills (Table 2).

**Table 2:** Adapted format for Gentile’s taxonomy of motor skills [17]

	Action Function			
	Body Stability		Body Movement	
Environmental Context ↓	No object manipulation	Object manipulation	No object manipulation	Object manipulation
Stationary regulatory condition and no-intertrial variability	1a	1b	1c	1d
	Practicing a golf swing without a club	Hitting balls at a driving range, from the same spot	Practicing the same dance steps	Practicing the same dance steps with a partner
Stationary regulatory condition and intertrial variability	2a	2b	2c	2d
	Practicing separate tai chi moves standing on the same spot	Hitting ball with cue from different locations on a billiards table	Practicing different dance steps on own	Practicing different dance steps with a partner
In-motion regulatory condition and no-intertrial variability	3a	3b	3c	3d
	Riding on a stationary exercise bike at a constant speed	Fishing while sitting on a boat and only casting once	Practicing lawn bowls delivery with a walking step without a ball	Returning a tennis serve from a tennis ball pitching machine
In-motion regulatory condition and intertrial variability	4a	4b	4c	4d
	Riding on a stationary exercise bike at different speeds	Fishing while standing on a boat; throwing cast out in a new spot each attempt	Walking in a busy shopping centre	Riding a bike on a bike path

As an example, when golf is broken down the first and most simple progression of the 16-item taxonomy will be practicing the motion of a swing (stationary) without a club or ball (no object), in an indoor driving range (closed environment) and then repeated (performance condition remaining constant). Progressions that increase the challenge will include the use of a club and golf ball (object manipulation), hitting a ball in new positions each attempt, such as playing on an 18-hole golf course (open environment and performance condition differing each trial). Further, adding object manipulation and a changing/open environment (e.g., wind, slope, grass, sand etc.) increases the challenge, but the golf shot is still taken in a stationary position. Other activities such as returning a tennis serve will require body movement in order to return the shot, again increasing the difficulty.

### Data analysis

FG discussions were recorded using an audio recording device (average duration was 77 min [range 65 to 105 min]) and transcribed verbatim. The qualitative data were generated using a grounded theory approach from FG content rather than solely from researcher generated questions [25]. First, becoming immersed in the data allowed embedded meanings and relationships to emerge [24]. Inductive enquiry was used in the early stages to generate new understanding from research participants’ perspectives [26]. The qualitative data management software NVivo (v10) (QSR International, 2013) served as a platform for ordering and forming inductive codes. These codes were sorted into groups (themes) in order to identify commonalities within data. The themes were then re-read and analysed to discover three major concepts. This process enabled the researchers to produce ‘conceptually dense’ data developing theory grounded in the participants’ views [25]. The final stages of analysis took a more comparative approach, involving careful examination of deviant cases [24]. Research team meetings were held to confirm coding analyses. Coding discrepancies were discussed and reconciled. This process was repeated until theoretical saturation and coding agreement was reached among team members.

Data analysis was based on an inductive exploration of older adults’ views and perceptions of physical activity types (ranging in complexity), with probing focusing on reasons participants did or did not participate in activities, rather than measuring aspects such as frequency counts of activities. Themes and concepts are presented individually in Table 3, however they are not separate entities. Similarly, responses shown in Table 4 are based on FG discussions and participant perceptions when rating the activities. Positioning activities on a list was used as a visual tool during the FGs to promote further conversation and comparison between activities. The initial two FGs were asked about difficulty and challenge but the order for challenge was not recorded as participants pointed to the order instead of saying the actual number (missing data is indicated by ‘-’ with 4/6 FGs having complete data for the second rating activity in Table 4). Comparisons and similarities across categories are explored in the results section. To maintain confidentiality, participants’ names are not reported. Instead, participants’ contributions are presented collectively, with reference to their gender and focus group (village = V and community = C). For example, MV1 represents a male participant from village 1 (see Table 4 for the associated focus group number). As participant identification numbers were not incorporated in the transcripts, in instances where two participants of the same gender and from the same FG speak one after another, the second participant will have the letter ‘a’ to clarify it was not the same participant (i.e., MV1a). This allowed further analysis across gender and focus groups.

**Table 3:** Resulting themes and concepts

Themes	Concepts
• Physical effort • Pace of activity • Age appropriate	Perceived difficulty
• Specific skill • Physical attributes • Environment influence • Level/ type of activity	Perceived challenge (Prompted to focus on skill involved and dismiss physical effort)
• Prior experience • Instructor/ teacher • Likelihood to participate	Lifetime participation (Preference of activities and influences)

**Table 4:** Participants rating activities for degree of difficulty and challenge on a scale from 1–10

FG Question	Easy vs. Difficult						Less challenging vs. most challenging					
Group/number	V1	V2	V3	C4	C5	C6	V1	V2	V3	C4	C5	C6
Very Easy	Walking	Walking	Walking	Fishing	Fishing	Walking	–	–	Walking	Walking	Walking	Walking
	Fishing	Swimming	Swimming	Walking	Walking	Fishing	–	–	Swimming	Fishing	Fishing	Cycling
Easy	Swimming	Lawn Bowls	Gym	Lawn bowls	Lawn Bowls	Dancing	–	–	Cycling	Swimming	Gym	Fishing
	Dancing	Golf	Aerobics*	Swimming	Golf	Cycling	–	–	Gym	Gym	Aerobics*	Dancing
	Gym	Dance	Golf	Golf	Swimming	Lawn bowls	–	–	Aerobics*	Cycling	Swimming	Lawn Bowls
Difficult	Aerobics*	Gym	Lawn Bowls	Aerobics*	Dance	Swimming	–	–	Dancing	Aerobics*	Cycling	Swimming
	Lawn bowls	Cycling	Dancing	Dance	Cycling	Aerobics*	–	–	Lawn bowls	Dancing	Dancing	Aerobics*
	Golf	Fishing	Fishing	Gym	Gym	Gym	–	–	Golf	Lawn bowls	Racquet sports	Gym
Most Difficult	Cycling	Aerobics*	Racquet sports	Cycling	Aerobics*	Golf	–	–	Fishing	Racquet sports	Lawn bowls	Golf
	Racquet sports	Racquet sports	Cycling	Racquet sports	Racquet sports	Racquet sports	–	–	Racquet sports	Golf	Golf	Racquet sports

Aerobics* = Aerobics and calisthenics

2/6 FG missing data indicated by ‘–’

## Results

There was a total of 36 participants (mean age ± SD, 76.1 ± 5.9) including seven male and six female participants in community groups, and four male and 19 female participants in village groups. Information for country of birth was given for 32 out of 36 participants; of those 20 (62.5 %) were born in Australia, 11 (34.4 %) in Europe and 1 in the Middle East. The results are presented under three main concepts: perceived difficulty; perceived challenge; and lifetime participation (Table 1).

### Perceived difficulty

#### Physical effort

First, it became clear that physical effort involved in a range of physical activity types was a key to how difficulty was perceived. Of the activities rated easy through to difficult, on a scale of 1-10, the only three in the very easy category among all groups were: walking, fishing and swimming (see Table 4). When asked, “Why is fishing the easiest?” MC4 responded, “Well there’s absolutely no physical effort.” Perceived physical effort was characterised by words, with easy activities identified as gentle (for example water aerobics, tai chi) and difficult activities as strenuous and vigorous, (such as racquet sports): “They’re vigorous – you’re bending, twisting. You’re using more of your body in racquet sports I think” (FC5). FC5a agreed: “same with your aerobics and calisthenics as well.”

#### Pace of activity

Activities where participants could work at their “own pace” or have a “breather” were considered as requiring less physical effort “…with racquet sports you either play at that pace or you don’t play at all. You can’t play a slow game” (FC4). This also relates to whether participants were able to anticipate what comes next. For example, dance was described as “more the casual - well not casual, but more even pace,” whereas the same participant (MC4), described aerobics as “…very very hectic, you know, very, very full on”, because they could not anticipate what would come next:

It comes down to whether you get any breaks or breathers – so in cycling, walking, aerobics and swimming there’s probably no space there for you to have a breather. Racquet sports would be the same thing. Dance the same. Golf you get a breather, lawn bowls you get a breather, and fishing you get a breather – so it just depends on how you do it yourself. A gym workout you can get a breather. I think though that’s pretty fair. (MC5)

#### Age appropriate

Ultimately, the age appropriateness of activities was an important factor. For example, five participants from village two showed interest in participating in aerobics and calisthenics. FV2, for example, said, “I think it’s [aerobics] something you’d try out or go along and have a look and then determine whether or not it was appropriate.” The V1 participants mentioned their village provides a modified aerobics class considered suitable due to “…aerobic/ calisthenics the exercise we do is quite gentle and [suitable] to our age group” (FV1). Vigorous activities or those requiring physical effort were considered less appropriate with some groups laughing or giggling when some activities were introduced. For example, when asked to rate weightlifting, FV3 said: “Oh [laughter] no way… That’s for the plumber’s son.” Furthermore, participants had a similar response to one another when running and jogging were mentioned: “No that is not for us” (FV1); [everyone shaking their heads in agreement], and FV2 responding, “Oh, no way” [most agree], “It’s a young person’s sport.” All FG participants, in contrast, considered tai chi appropriate: “Yes that’s for old people” (FV1) and easier “because it is very gentle” (FV1a). Alternatively for water aerobics some participants did not assess themselves as old enough yet. When asked if they would give it a go FC5 replied: “Yes I would. Not at my age, but if I get to 80 I would. I’ve done it and I like it and I’d do it again.”

As shown in Table 4, pace and physical effort influenced perceptions of difficulty. Although physical effort and pace were not the only concepts recognised, with FG C6 rating golf in the most difficult category, and some groups recognising lawn bowls and fishing in the difficult categories. These concepts are further combined with discussion points that rated activities based on skill, using the same scale from 1-10, moving from less challenging to most challenging (disregarding physical effort).

### Perceived challenge

Perception of difficulty in relation to participating in a range of activities was firstly based on cardiovascular and physical effort. Once prompted to think about the skills involved and ignore physical effort, a different picture developed.

#### Specific skills

It is clear that participants recognised activities as having skill components, increasing challenge in performance. “I think golf is way ahead. It’s difficult. So many factors involved in golf. You have to have a perfect technique and nobody has a perfect technique” (MC4). Participants were also able to recognise key concepts. Whether an individual is stationary while performing the skill or moving, and whether an individual interacts with an object, were central to definitions of what was challenging. Comparisons are given for yoga and dancing which recognise body stability. With yoga “…you’re in one place” (FV2), whereas dancing involves body movement “…with the dancing one, you are moving, jumping around more. So that’s why I’d bring in that differentiation…” (FV2a). Furthermore FC5 stated:

Yes but racquet sports are a bit different. You have to be where the ball is to return it, but with gym you can be up and down on the one spot. Yeah and you can go at your own pace depending on what sort of – the degree of difficulty it’s going to be.

Participants identified equipment as contributing to the challenge of an activity.

I played indoor bowls… The skill is on the one side the ball has different weight and has rings on it, and you have to know if you want to come in from this side or come in from that side. So it depends how you hold the ball in your hand, and the ball itself. (MC6)

#### Physical attributes

Participants identified physical attributes including: balance; flexibility; co-ordination; aerobic fitness; rhythm; control; using the whole body; and muscle strength and endurance. These attributes influenced their perception of difficulty, some of which provided too much challenge for participation. Flexibility and balance were reoccurring attributes: “you do have to be able to stand still, or you would fall over - balance is very hard in tai chi…” (FV3). When asked “So, what aspects with yoga might stop you from doing it? Interest?” FV2 responded: “I would find it now too challenging. My body isn’t flexible enough…”

#### Environment influence

Participants recognised the influence of different environments on difficulty in performing an activity such as cycling: “if you are going up very steep hills then it becomes much more difficult than if you are just going to cycle around a flat part or something” (FV1). Furthermore, a changing environment was recognised as providing different challenges: “…I suppose fishing from a boat has got to be different to fishing from a beach” (FC4). FC4 further explained reasons why it would increase challenge: “you can change where you fish, and I suppose the skill set you have has to be adjusted to where you are” (FC4). Adjusting skill sets depending on the environment was discussed in greater detail in relation to golf: “you have to judge the course, speed of ball, speed of green, what balls to use” (FC5) which also implies the use of cognitive engagement. FV2 made a similar assessment related to lawn bowls:

There’s a certain amount of skill though, you’ve got to judge your bowl versus the wind, then everything else and getting it down and getting the right bias and speed. There is a fair amount of challenge in it… I’m only a learner and on my P plate [i.e., second phase of one’s driving license, following a learner’s permit], off the L plates. (FV2)

Environment was also discussed in relation to weather conditions which affect the choice of activities by participants in one FG; hot summer days were highlighted as being too warm for activities such as walking whilst participants were less inclined to swim during winter: “It’s seasonal” (FC5). When asked, “…what would stop you going?” FC5a responded, “Only the winter, cold weather.”

#### Type and level of activity dependent

A common theme recognised by participants was that “It depends on what level you’re going to do these things” (MC4). This is further explored in FV2’s statement:

Once again it all depends on the level, because there’s simple tai chi and then you’re looking at tai chi with swords and all that, it was really a martial art. So whether it’s ahead of walking is debatable. Like all of those sports, it depends on the level.

This was a common theme further explored in relation to the dance style, “depends what dancing it is” (FV3), and dance type: “you dance yourself, and you have fast dancing, slow dancing, traditional dancing… You may be dancing with a partner, and it’s very social in that regard…” (MC4). Some styles were considered less appropriate than others: “line dancing, imagine us doing line dancing. That’s out of the question for us… waltzing or the jazz waltz or something like that, that’s different…” (FV3), implying these styles are better suited for the participants’ age group.

### Lifetime participation

#### Prior experience

Prior experience influenced willingness to try an activity and participate:

…if you come to me tomorrow and say “we’ll go to [the city] and do some Aerobics” and I’d say “well you can I will go on the golf course and hit a golf ball.” I’m just not into that. It’s shyness. You just don’t feel like putting your shorts on or whatever, your outfit; you just won’t feel comfortable. That’s what I think. (MC6)

For tai chi, perception of difficulty differed for the female participant who had prior experience, “Oh tai chi is very easy. It’s good for the memory. Once you’ve learned, you can start it anytime” (FC6). A male participant, MC6 in contrast, was more hesitant in his first response pointing to a seven, rating tai chi in the difficult category, but FC6 disagreed: “it’s not that hard, no.” FC6’s prior experience in tai chi may have influenced her view, whereas the male participant when re-asked, “You think it’s a bit more difficult, tai chi?” MC6 replied, “I think so, but you know I’ve never done it before.” These findings show prior experience may influence how an activity is perceived.

MV1: Yeah, well so if you are [a] golfer you would probably put it another one [rating easiest to difficult 1-10 scale]. I am not a golfer. It comes down to -

Moderator: So you think a lot of these activities depend on your own experience?

Participants: Yes, yes [all nodding heads].

FV1: I mean I started riding my bike when I was 6 years old. So for me it’s not very difficult… It would be if I was starting now.

FV2 further discussed an association between less challenging and previous experience for dance: “I don’t know there’s a lot of challenge except when you start learning, and most people have learned some sort of dancing in their younger years, even if it’s at school.” Not having prior experience was also considered a psychological barrier for MC6:

I think the most thing is when you get a certain age like we are, if someone says “come tomorrow to do aerobic[s]” and say you’ve never done it, you might be embarrassed, because for a start you stand there like an ox and then there’s music and someone says 1, 2, 3 and off you go and you just don’t have the chance…

#### Instructor, teacher, personal trainer

Positive relationships with instructors also influenced participation. While the discussion guide did not focus on questions in relation to instructors and teachers, participants stated the importance of a good instructor: “you have to have the right teacher” (MC6). When participants from V2 were asked if they would be interested to participate in gym workouts, all eight participants agreed, “Yes, I’d give it a go” (FV2). The moderator explored further whether any barriers would prevent participants from continuing their participation in gym workouts:

Yes, and whether or not with someone who’s teaching you, you develop a rapport with that teacher, so that the teacher was actually reacting to the feedback she got from the class too and was grading the classes appropriately. (FV2a)

#### Likelihood of participation

While all participant groups identified as having participated in a range of physical activities throughout their lives (see Appendix C), the range of physical activities declined from 30 years of age and onwards. When asked about preference of physical activities in terms of long-term participation, four themes were discovered to be important including social environment, enjoyment, ill health and self-interest. “The most important thing is social” (MC4). Having a positive social environment also helped keep FC4 “…in contact with other people. [My husband] is only semi-retired, so it gives you a whole new range of people.”

Physical activities that were perceived as being enjoyable increased the likelihood that participants would give it a try. “I used to see that team of people doing tai chi and they looked as if they were enjoying it, and it looked interesting, so I’d like to try it” (FC4). Rather than focusing on competition, enjoyment was the focus:

FV1: No we don’t want to be the best.

FV1a: We just want to do something.

FV1: No more competition. We don’t want competition.

A number of ill health factors were stated as barriers: “My health wouldn’t allow it – I can’t” (MC5). Positive health outcomes were motivating factors to continue participation: “We are at an age now where we have to keep ourselves from deteriorating, from getting sick and everything. We’re getting older” (FC5).

Furthermore, self-interest and “…whether it appeals to somebody” (FV2) influenced ongoing participation: “…There may be a bus to take you at the door, but you may have no interest in it, not necessarily a physical side to it, but it just doesn’t appeal.”

Overall, the manner in which older adults perceived difficulty and challenge involved in a variety of activities was similar between community and village groups. It became evident within all FGs that activities cannot be thought about in a context-free sense. Social and interpersonal issues, such as embarrassment, rapport with instructors, prior experience and familiarity, in addition to physical effort, were other central features of older adults’ perceptions of physical activities.

## Discussion

This is the first study to qualitatively analyse older adults’ perceptions of the motor challenge and difficulties involved in performing different types of physical activities. From older adults’ perspectives the main findings suggest physical effort and pace underpin perceptions of increased difficulty. However, when prompted, a variety of movement characteristics were identified as increasing challenge with the main aspects mentioned being changes in environmental conditions and using an object.

### Participants’ understanding of Gentile’s taxonomy- the match and mismatch

Participants acknowledged that when activities are changed or modified it can increase or decrease the degree of complexity. While they did it on a descriptive level by comparing between activities (e.g., more skill in golf vs. racquet sports) or within activities (e.g., fishing on surface or from a boat), in doing so they acknowledged Gentile’s logical sequence to break up the movement challenge [17]. For example, Gentile’s creates subdivisions for environment context (closed versus open) and for performance conditions (remain constant or change), whereby the second level is always more challenging.

Other variations in performing the activity were also discussed in FGs, some of which reflect Gentile’s action function (i.e., body stability and object manipulation). Participants’ perspectives gave a different insight. Some participants identified objects as increasing challenge, although dancing with a partner (object manipulation) was not viewed as increasing challenge, instead was perceived as making the activity more social. While Gentile’s considers body stability as more simple than body movement, for older adults this may not be simple enough.

While Gentile’s offers a versatile platform, pace of an activity recognised as activity intensity in the physical activity and public health domain, is not considered in the taxonomy. Suitable progressions particularly for new participants regarding pace/intensity have been recommended by others [27] and is a key component in the ACSM’s exercise recommendations [8]. Greater sensitivity in Gentile’s classification is needed when applying it to older adults due to declining physical capacities, health status and senses (hearing and vision), as depicted in FGs by older adults wanting to participate in “gentle” and “age appropriate” physical activities. If pace/intensity is not adapted into Gentile’s it should be considered in conjunction with Gentile’s.

### Increase participation in CMS

Feelings of embarrassment or discomfort related to a lack of ability were described as barriers to learning a new activity or skill, especially among older males. This finding contradicts past research based in Canada showing a lack of skill in older adults (65+) as a stronger barrier among women (40 % strongly agreed) compared to men (18 % strongly agreed) [28]. Regardless, it highlights gendered and cultural variations [29] as barriers to participation in new physical activities and skills which should be considered in future intervention-based research.

In order to successfully learn a new skill, ensuring the task difficulty level is suited to an individual’s ability is recommended [16]. The changing task model considers reasons that may mean an individual has to alter the way he/she performs a skill. Often based on biological development with great emphasis on puberty and injury [18, 30], greater knowledge and teaching platforms should centre around ill health or regression (physical and cognitive) due to ageing. The changing task model in conjunction with Gentile’s could assist in providing progression for pace of activities. Findings here show instructors play an important role in modifying an activity to suit participant abilities and in encouraging continued participation. The utilization of a taxonomy such as Gentile’s can assist instructors in thinking through task demands systematically and thoroughly prior to establishing lesson plans, as recommended for teachers working with younger populations [31]. Adoption of such a tool could allow instructors (or researchers) to also focus on how best to progressively introduce cognitive load or balance in activities (or in future interventions).

While teaching fundamental movement skills is recognised as vital in developing and maintaining basic motor skills for children and adolescents, there are limited interventions in the field of acquisition and retention of sport skills in older adults. For example, in two systematic reviews most physical activity interventions for older adults either offered walking programmes or exercises that focus on single or multiple dimensions of fitness (i.e., strength, aerobic fitness and balance) [32, 33]. Few interventions offered mastery in motor skills in old age such as tai chi, swimming or dance [34].

For continued participation over the life course, activity type is a draw card irrespective of pace and intensity. Empirical evidence among women found that not all higher intensity activities (e.g., swimming, biking), declined in participation. While total amount of physical activity among women declined in the two age periods 55 to 64, and 75 or older, the distribution of popular activities did not vary much with age [35]. A significant finding in the FGs was that while participants identified some activities as more challenging than others, this did not hinder participants’ willingness to participate (with the exception of golf among some participants). Furthermore, participants expressed a stronger likelihood of considering an activity if they had prior experience, even if their experience was in their adolescence in line with findings from others [36, 37]. Therefore, to increase the likelihood of increasing participation in CMS, a greater variety of age appropriate activities particularly those that the older generation may have previous experience in (such as dancing and swimming) need to be offered.

As with children, motor skill acquisition has been implemented in physical education classes to achieve long-term sustainability. Applying a developmental perspective to physical activity participation has been previously suggested in the context of ageing [38], merely in relation to life transition such as becoming a retiree, informal caregiver, or intergenerational interventions. Here, we suggest that to enhance motor skills in old age, more challenging physical activity programs should be introduced for those who are already in middle age in order to achieve transfer of skills and long-term retention across the life course.

### Strengths and limitations

To the best current knowledge, no study has examined older adults’ views on complexity and challenge in a range of physical activities. The main strength of this study lies in its use of qualitative focus groups to allow older adults to openly discuss a range of factors influencing their perceptions of difficulty and challenge in leisure and sport participation, and how to overcome such factors to increase participation through the developmental life stages. Findings, however, are limited due to the lower ratio of male to female participants in village focus groups, which is representative of population living in assisted accommodation [39]. This may have constrained the extent to which men contributed in these groups. However, probing questions were employed to ensure that diverging perspectives were captured within the focus groups data. Although we had 54 % male participants in community groups compared to 17 % in the village groups no variations were found between groups, therefore comparisons of data between genders were deemed robust. In this study, saturation was achieved (no new themes arose in the final focus group discussions), however whether unique themes influence males living in villages is a subject that needs further exploration. While this study has a reasonable sample size for a qualitative study, the characteristics of participants are limited to urban populations, with most participants identified as either Australian or European. As exercise preferences vary somewhat across cultures, ethnicity and place, our findings may not be generalizable. This highlights a need for further research with larger and more diverse samples [40–42].

## Conclusions

Results suggest older adults show interest in participating in a range of challenging activities – if offered. However, suitable progression is important. If the skill, ability and pace of an activity is not suited to the participant, particularly when starting out, older adults may discontinue participation, to avoid injury or embarrassment.

Movement science and public health disciplines should work together in developing learning platforms suitable for older adults at any developmental level (i.e., declining vision, neuromotor and cognitive capacity). One way is to incorporate specific health implications and consider the pace/intensity of an activity into the taxonomy, which has promise as a practically oriented tool, although it will require a developmental stage for instructors to become comfortable in applying this framework to older adults. Given that CMS can have benefits in the area of balance, injury prevention and possibly coordination, a greater focus should be placed on increasing participation amongst older adults.

## Abbreviations

ACSM: American College of Sport Medicine
CMS: Complex motor skills
